# Translating the epitranscriptome

**DOI:** 10.1002/wrna.1375

**Published:** 2016-06-27

**Authors:** Thomas Philipp Hoernes, Matthias David Erlacher

**Affiliations:** ^1^ Division of Genomics and RNomics, BiocenterMedical University of InnsbruckInnsbruckAustria

## Abstract

RNA modifications are indispensable for the translation machinery to provide accurate and efficient protein synthesis. Whereas the importance of transfer RNA (tRNA) and ribosomal RNA (rRNA) modifications has been well described and is unquestioned for decades, the significance of internal messenger RNA (mRNA) modifications has only recently been revealed. Novel experimental methods have enabled the identification of thousands of modified sites within the untranslated and translated regions of mRNAs. Thus far, N ^6^‐methyladenosine (m^6^A), pseudouridine (Ψ), 5‐methylcytosine (m^5^C) and N ^1^‐methyladenosine (m^1^A) were identified in eukaryal, and to some extent in prokaryal mRNAs. Several of the functions of these mRNA modifications have previously been reported, but many aspects remain elusive. Modifications can be important factors for the direct regulation of protein synthesis. The potential diversification of genomic information and regulation of RNA expression through editing and modifying mRNAs is versatile and many questions need to be addressed to completely elucidate the role of mRNA modifications. Herein, we summarize and highlight some recent findings on various co‐ and post‐transcriptional modifications, describing the impact of these processes on gene expression, with emphasis on protein synthesis. *WIREs RNA* 2017, 8:e1375. doi: 10.1002/wrna.1375

For further resources related to this article, please visit the WIREs website.

## INTRODUCTION

Messenger RNA (mRNA) translation is a central process in every living organism. The assembly and operation of the translation machinery are very costly and can consume up to 40% of the cellular energy.^1^ Therefore, protein synthesis needs to be strictly regulated in many aspects. The regulation of translation is typically associated with the necessity of regulatory proteins and regulatory non‐coding RNAs (ncRNAs). However, equally important for the translation process are nucleotide modifications, which are present in all involved classes of RNA. Ribosomal RNAs (rRNAs), transfer RNAs (tRNAs) and mRNAs are co‐ or post‐transcriptionally modified. Whereas the precise function of many of these nucleotide derivatives remains enigmatic, it has become evident that many of those are important factors for numerous biological processes, such as ribosome assembly,^2^ mRNA stability,^3^, ^4^ RNA folding,^5^ and accurate and efficient protein biosynthesis,^6^, ^7^ to name a few. More than 100 different types of RNA modifications in almost every class of non‐coding and coding RNAs have been reported.^8^

Most of the modifications described thus far have been identified in tRNAs.^8^ These modifications strongly vary in chemical and structural complexity and are necessary for the proper folding and function of tRNAs. Several reported modifications are crucial for the correct geometry of the anticodon loop and therefore affect the decoding process.^6^, ^7^ Other modifications are mandatory for the aminoacylation of the respective tRNA body.^9^ However, many tRNA modifications are assumed to have no or only a minor impact.^10^ Considering the effort necessary to specifically introduce modifications, the functional role of these modifications during the ‘life cycle’ of a tRNA might not yet be revealed.

The second class of RNA that requires modification for functionality is ribosomal RNA. The number of rRNA modifications identified in prokaryotic organisms is rather small (23 modifications in *Thermus thermophilus* 11 and 35 modifications in *Escherichia coli*)^12^, compared with eukaryotes (~100 modifications in yeast and 200 modifications in vertebrates).^13^ Most of the modified nucleotides are located near the peptidyl transferase center in the large ribosomal subunit and the decoding site in the small ribosomal subunit. The function of most modifications is obscure.^11^ Whereas only deletions of whole clusters of rRNA modifications severely impair the translation capability of ribosomes, the loss of single RNA nucleotide derivatives has a rather small effect on the basic steps of translation.^14^ Even ribosomes carrying rRNAs without any post‐transcriptional modifications are capable of synthesizing full‐length proteins *in vitro*, suggesting that these RNA modifications are not fundamental for all basic steps of protein biosynthesis.^15^ However, some methylated nucleotides have been implicated in fine‐tuning translation initiation and decoding fidelity,^14^ and several pseudouridines (Ψs) are pivotal for forming the intersubunit bridge B2a.^16^ Still many questions concerning the role of rRNA modifications during protein synthesis or ribosome assembly remain unanswered.

Although the co‐ and post‐transcriptional modification of mRNAs had been described decades ago, recent computational approaches and high‐throughput RNA sequencing techniques have revealed thousands of novel modification sites within coding sequences and untranslated regions (UTRs) of mRNAs.^17^, ^18^, ^19^, ^20^, ^21^, ^22^ These findings have boosted interest in the types and potential roles for mRNA modifications during gene expression.

Post‐transcriptional modifications of RNA can be historically classified into two groups: edited RNA and modified RNA. RNA editing is usually understood as posttranscriptional RNA processing (except capping, splicing and polyadenylation) that changes the RNA nucleotide sequence compared with the genetically encoded sequence. This processing can be achieved through the insertion/deletion of nucleotides or deamination of nucleobases, generating either standard nucleotides or the rare nucleotide inosine (I).^23^ mRNA modifications, however, are considered alterations in the chemical composition or conformation of a nucleotide that potentially influences the function or stability of the transcript. The definition of edited or modified RNAs should not be taken too strictly, as these terms are often context‐dependent. In the 1970s, internal *N*^6^‐methyladenosine (m^6^A) and low levels of 5‐methylcytosin (m^5^C) were revealed in mRNAs of eukaryotic cells.^24^ Since then, other RNA nucleotide derivatives, such as are Ψ^17^, ^19^, ^20^ and *N*^1^‐methyladenosine (m^1^A),^21^, ^22^ have been reported within mRNAs. Whereas most of the nucleotide derivatives were found in eukaryotic organisms, some derivatives were also abundant in prokaryotic mRNAs.^18^, ^25^

The modification and editing of mRNAs are essential processes that influence and regulate gene expression at the post‐transcriptional level. In this review, we summarized and highlighted important findings in this field. mRNA modifications are involved in many aspects of mRNA processing, stability, folding and translation. We also specifically focus on the involvement of mRNA modifications in protein synthesis, and discuss the impact of these processes on gene expression.

## mRNA EDITING DIVERSIFIES PROTEIN SYNTHESIS

### Nucleotide Insertions and Deletions

In 1986 Benne and co‐workers first described striking discrepancies between the DNA sequence of a gene and the RNA sequence of the corresponding transcript.^26^ The authors revealed four uridines within the mRNA of the mitochondrial oxidase II subunits in trypanosomes that were not genetically encoded. This observation implied that nucleotides are inserted into the mRNA during or after transcription, thereby repairing a genomic frameshift site.^26^ In subsequent studies, more examples of U insertions and deletions were identified, and it became evident that these editing processes are characteristic for the order of kinetoplastid protozoa.^27^ Indeed, the post‐transcriptional insertion of uridines into the transcripts of certain mitochondrial genes can be rather extensive,^28^ making it challenging to identify the corresponding DNA sequence.

**Figure 1:**
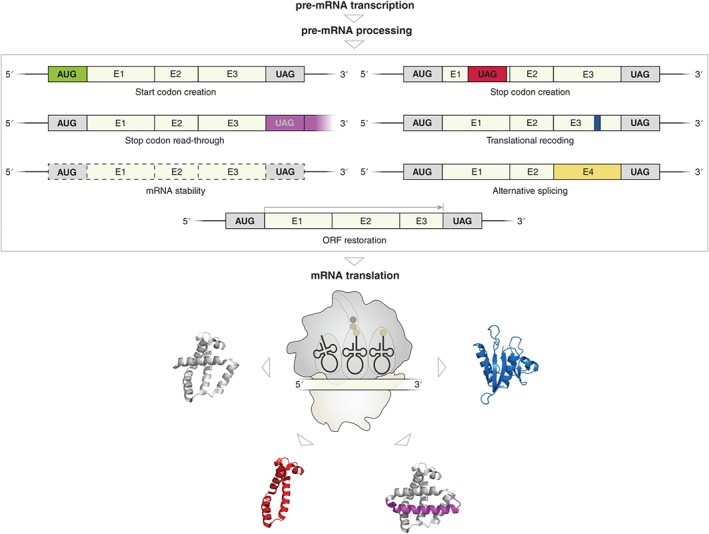
Schematic representation of mRNA editing and its effect on translation. Editing of pre‐mRNA transcripts can generate start codons (green) and stop codons (red) by insertions of nucleotides or by base conversions. Base conversions potentially remove stop codons causing a prolonged open reading frame (purple). mRNA editing in the coding sequences can lead to non‐synonymous codon substitutions (blue). In addition, editing within the coding sequences or in the 3′ UTR of the mRNA can induce alternative splicing (yellow) and altered mRNA stabilities (dashed frame), respectively. Insertions or deletions of nucleotides can cause a restoration or even a creation of an ORF (gray arrow). Edited mRNAs are subsequently subjected to translation and result in shortened/extended protein products (red and purple, respectively) or functionally altered proteins (blue) (E: exon; ORF: open reading frame).

In addition, guanosines (Gs) and adenosines (As) are also inserted into mRNAs of the Paramyxoviruses and the Ebola viruses, respectively.^29^ The mitochondrial mRNAs of *Physarum polycephalum* harbor co‐transcriptionally inserted cytosines (Cs) and even various dinucleotides (AA, CU, GU, GC and UA).^29^, ^30^ Independent of the number or type of post‐transcriptionally inserted/deleted nucleotides, the genetic information can be revised co‐ and post‐transcriptionally, thereby generating open reading frames (ORFs) through the creation of start and stop codons. In addition, the reading frame can be changed and the sequence information of the mRNA altered, thereby significantly impacting gene expression (Figure 1).

### C‐to‐U Editing

In addition to the insertions/deletions of nucleotides, the message can also be revised by changing the identity of RNA nucleotides. These changes are achieved through the enzymatic alteration of the chemical composition of nucleobases, resulting in a new nucleotide identity considered as a nucleotide substitution.

The first example described was a C to U substitution within the ORF of apolipoprotein B (apoB).^31^, ^32^ Apolipoproteins are essential components for lipid transport and lipid metabolism. ApoB primarily exists in two isoforms: apoB100 and apoB48. In humans, apoB100 is synthesized in the liver as an essential component of very low‐density lipoproteins (VLDL), intermediate‐density lipoproteins (IDL) and low‐density lipoproteins (LDL). ApoB48 is expressed in the small intestine and is present in chylomicrons and their remnants.^33^ The determination of the mRNA sequence of intestinal apoB revealed the post‐transcriptional substitution of a C with a U in the CAA codon, resulting in an UAA stop codon. The editing of the mRNA therefore leads to a truncated protein product, i.e., apoB48, with distinct functions compared with full‐length apoB100. The responsible cytosine deaminase complex required for editing apoB mRNA is APOBEC‐1 together with RNA‐binding auxiliary protein APOBEC‐1 complementation factor (ACF) and the RNA‐Binding‐Motif‐Protein‐47 (RBM47) (reviewed in Refs 34, 35, 36). Through the identification of the editing complex a conserved RNA motif, the mooring sequence, has been revealed, which recruits the cytosine deaminase to the editing target. The identification of the conserved mooring sequence led to the discovery of additional mRNAs, such as the oncogene neurofibromanin 1 (NF1), that are edited in humans.^35^

Thus far, C‐to‐U editing has only been observed in eukaryotes, but not in bacteria and archaea. C‐to‐U editing is highly prominent in plants. With only few exceptions, the mitochondrial and plastid mRNAs of all land plants show editing.^37^

C‐to‐U editing is certainly not restricted to the coding sequences of mRNAs. Several editing sites have been detected, particularly in the 3′ UTRs of mRNAs, which therefore do not alter the amino acid sequence of the resulting product.^38^, ^39^ Thus far, it is not clear how these editing sites influence gene expression. It is feasible that altered sequences modulate the efficiency of the translation process, alter RNA‐protein binding affinities and consequently regulate mRNA translation.^38^ In addition, miRNA target sites could be affected as described for A‐to‐I editing.^39^, ^40^

In addition to the C‐to‐U editing, substitutions of U with C were observed in land plants and mammals.^29^ For example, in the Wilms tumor gene (WT1), encoding a zinc finger transcription factor, the U‐to‐C conversion results in an exchange of a leucine with a proline in the final protein.^41^ Although these editing events have been identified in rats, mice and humans, their functional roles, the editing mechanism itself and the executing enzymes remain elusive.^41^, ^42^, ^43^

Recently, also G‐to‐A editing has been described for the WT1 mRNA^44^ and the mRNA of human tryptophan hydroxylase (TPH).^45^ Thus far, little is known about the role and impact of the edited sites on the enzymatic activity of the synthesized protein. With the rise of high‐throughput sequencing technologies, more examples will likely be revealed. Whether these substitutions add to the list of mRNA modifications that alter gene expression or turn out to be sequencing artifacts should be carefully evaluated in future studies.^46^, ^47^

### A‐to‐I Editing

The conversion of adenosine to inosine (I) is the most prevalent form of RNA editing. More than 100,000,000 editing sites were computationally predicted within the human transcriptome.^48^ Chemically, the process involves a hydrolytic deamination at the C^6^ position, resulting in the conversion of A to the rare nucleotide I. The substitution of this amino group as a hydrogen donor with a carbonyl‐oxygen as a hydrogen acceptor generates a similar Watson–Crick edge as G. Therefore, this type of editing is also occasionally referred to as A‐to‐G editing.^49^ Consequently, editing within double‐stranded RNA results in an I‐U mismatch, and the translation machinery recognizes I as a G instead of an A, potentially resulting in an amino acid substitution.

The enzymes responsible for the deamination reaction are adenosine deaminases acting on RNA (ADARs). These enzymes are highly conserved across metazoans,^50^ but the number of genes and isoforms varies between different species (reviewed in Ref 51). In mammals, two catalytically active ADARs have been described: ADAR1^52^ and ADAR2.^53^ A third member of the ADAR‐family has been identified, i.e., ADAR3, but the catalytic function of this enzyme has not been demonstrated.^54^ ADAR1 and ADAR2 are expressed in a wide range of tissues, whereas ADAR3 is exclusively expressed in the brain.^55^ All ADARs have an N‐terminal double‐stranded RNA‐binding domain (dsRBD) and a C‐terminal deaminase domain in common. Therefore, double‐stranded RNA regions of mRNAs^56^, small RNAs^57^ and viral RNAs^58^ are targets for A‐to‐I editing. In mammals, ADARs are essential for development,^56^, ^59^ and altered A‐to‐I editing of various RNAs has been associated with a wide range of diseases, such as Alzheimer's disease or amyotrophic lateral sclerosis.^60^, ^61^

In the human transcriptome, more than 99% of the editing sites are reported to be positioned in Alu sequences, which are short interspersed nuclear elements (SINEs).^48^ Millions of these repeat sequences have been identified in the human genome, and these sequences are particularly concentrated in gene‐rich regions.^62^ Two repeat sequences are frequently observed in close proximity to each other, forming long double‐stranded regions representing ideal targets for the editing machinery. The role of Alu sequence editing is currently being investigated. Alu sequence editing has been associated with enhanced degradation through RNase III Tudor staphylococcal nuclease (Tudor SN) activity,^63^ altered RNA structures, mRNA splicing^64^ and RNA‐protein binding affinities.^65^

Although infrequent, the editing of protein‐coding sequences dramatically affects the protein product. The interpretation of I as G by the translation machinery can lead to non‐synonymous substitutions that significantly alter the function or activity of the protein products. The AMPA (alpha‐amino‐3‐hydroxy‐5‐methyl‐4‐isoxazolepropionic acid) glutamate receptor GluR‐B is the first RNA substrate identified.^66^ Editing causes the substitution of the CAG codon, encoding glutamine, to a CIG codon, which encodes arginine. This amino acid exchange dramatically affects the Ca^2+^ permeability of the AMPA receptor.^56^ About 99% of the primary transcripts undergo editing at this position and therefore the vast majority of GluR‐B subunits contains Arg but not the genetically encoded Gln. Mutational studies have shown that mutant mice harboring Gln instead of Arg die within weeks after birth.^67^ Another RNA transcript that undergoes RNA editing is the mRNA of the serotonin receptor 5‐HT_2C_. A total of 5 positions are edited, and these alterations affect the activity of the receptor as a result of altered receptor:G‐protein coupling.^68^ In addition to these well‐known representatives of A‐to‐I edited mRNAs, other examples of this type of editing have been identified in mammals, *Drosophila melanogaster* and viruses.^69^, ^70^

Not only can A‐to‐I conversions change the genetic code and thereby influence gene expression, A‐to‐I editing even regulates regulatory small RNAs, such as miRNA. Numerous effects of A‐to‐I editing on the functions of miRNAs function have been reported. The editing alters pri‐miRNA biogenesis, miRNA expression and miRNA selectivity (reviewed in Ref 70). In addition, the miRNA target undergoes RNA editing, thereby altering the miRNA target sequence and consequently modulating miRNA‐mediated regulation.^71^

The effect of RNA editing on gene expression and particularly translation has been well investigated. Insertions/deletions generate ORFs through the creation of start and stop codons within existing ORFs and nucleotide substitutions through deamination alter the codon identity, thereby affecting the amino acid sequence (Figure 1). Far less is known about internal mRNA modifications, such as m^6^A, Ψ, m^5^C and m^1^A, which are abundant in coding sequences and the UTRs of mRNAs. Upon first sight, some of these modifications are not likely to significantly alter the base pairing characteristics or the stability of the modified mRNA. Nevertheless, recent studies have reported many unexpected aspects that are influenced through mRNA modifications, revealing them as important factors that regulate gene expression.

## mRNA MODIFICATIONS REGULATE TRANSLATION

### *N* ^6^‐methyladenosine

In the 1970s, *N* ^6^‐methyladenosine (m^6^A) was among the first post‐transcriptional modifications reported as abundant at high levels within mRNAs.^72^, ^73^, ^74^, ^75^, ^76^ The m^6^A modification has been identified in the mRNAs of eukaryal organisms ranging from yeast and plants to mammals.^72^, ^77^, ^78^, ^79^, ^80^, ^81^ Recently m^6^A has also been described as a naturally occurring mRNA modification in bacteria.^25^ Within eukaryotes m^6^A is the most abundant internal mRNA modification, accounting for 0.1–0.5% of all As (m^6^A/A),^72^, ^82^, ^83^, ^84^ which translates to approximately three m^6^A residues per mRNA.^81^

The precise location of m^6^A within transcripts is debated, primarily because initial techniques could not map m^6^A at single‐base resolution.^85^ The established high‐throughput sequencing approaches are based on m^6^A‐specific antibodies, as m^6^A does not affect base pairing and is not prone to chemical modifications that would facilitate detection, enabling refined mapping and detailed quantifications.^81^ m^6^A is enriched in regions in direct proximity to stop codons, in long exons and transcription start sites.^86^, ^87^, ^88^ Owing to the cross‐reactivity of the antibody with *N* ^6^,2′‐*O*‐dimethyladenosine (m^6^Am), it is feasible that especially hits in the vicinity of transcription start sites also derive from m^6^Am, which is part of the 5′ cap.^89^ However, the overall methylation pattern of transcripts was found to be conserved in mammalian cells. Several groups have shown that the methylation topology is preserved in embryonic and somatic cells of humans and mice.^88^, ^90^, ^91^ In addition, a consensus motif for the introduction of m^6^A (Pu[G>A]m^6^AC[U>A>C]; Pu = purine) has been proposed, but only a fraction of the consensus sequences actually harbors m^6^A.^81^, ^92^

In yeast m^6^A is induced during meiosis,^77^, ^93^ indicating that the introduction of m^6^A might not only be cell type‐dependent but also dynamic during the cell cycle and development.^87^, ^90^, ^91^

**Figure 2:**
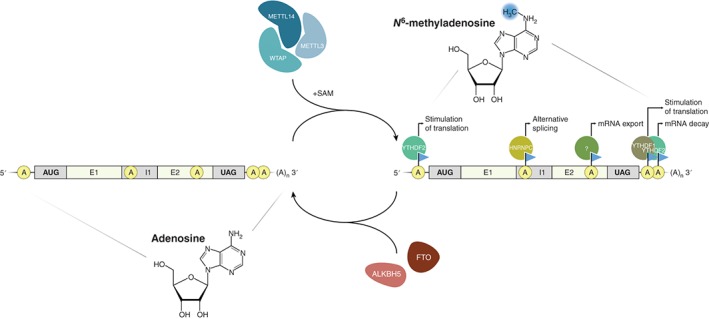
The dynamics of the m^6^A methylome. A METTL3‐METTL14‐WTAP methyltransferase complex (blue) mediates adenosine‐to‐m^6^A conversion of mRNAs.^95^, ^96^, ^97^ Once deposited, m^6^A fulfills distinct functions dependent on its localization within a transcript and the reader proteins (green) interacting with the m^6^A mark (blue triangle). m^6^A positioned within UTR sequences stimulates translational initiation via YTHDF1 or YTHDF2.^98^, ^99^ Alternatively, an YTHDF2‐m6A interaction in the 3′ UTR also induces transfer of mRNAs to decay sites.^4^ Other reader proteins affect alternative splicing^100^, ^101^ or processing and nuclear export of mRNAs.^102^ Eraser proteins, i.e., FTO or ALKBH5 (red), dynamically demethylate m^6^As^83^, ^84^ (E: exon; I: intron).

Another layer of complexity and dynamics is added by the finding that these methylations are reversible, making m^6^A unique between other thus far described modifications.^81^, ^84^, ^94^ The dynamic methylations and demethylations of A are mediated through distinct sets of proteins that have been rather well characterized. These enzymes can be divided into (1) m^6^A ‘writers’, which deposit m^6^A modifications, (2) m^6^A ‘erasers’ that catalyze the removal of m^6^A from the transcripts, and (3) m^6^A ‘readers’, which mediate the downstream effects of this distinct mRNA modification (Figure 2).

The first ‘writer’ described is METTL3 (methyltransferase‐like 3), a 70 kDa protein, functioning as a methyltransferase within a multi‐enzyme complex.^95^ Subsequently, METTL4 and METTL14 were bioinformatically identified, of which METTL14 has been biochemically validated to directly interact with METTL3, forming a large 1 MDa heterodimeric methyltransferase enzyme complex (Figure 2).^96^, ^103^ However, METTL3 and METTL14 both independently deposit m^6^A on transcripts, but show enhanced methylation activities *in vitro* and *in vivo* when combined.^96^, ^104^ The m^6^A writer complex is joined by WTAP (Wilms’ tumor 1‐associating protein), which itself does not exhibit methyltransferase activity, but might be crucial for the localization of the complex.^96^, ^97^

The first ‘eraser’ identified is the demethylase FTO (fat mass and obesity‐associated protein), which catalyzes the reversion of m^6^A to adenosine (Figure 2).^83^ The reaction proceeds via two labile intermediates, *N* ^6^‐hydroxymethyladenosine (hm^6^A) and *N* ^6^‐formyladenosine (f^6^A), whose biological functions remain elusive.^94^ A second m^6^A demethylase was identified in mammals, namely ALKBH5. This enzyme does not form intermediates and directly converts m^6^A to A.^81^, ^84^

Whereas m^6^A writers and erasers have attracted interest in the past, because of the compelling dynamic nature of the m^6^A landscape and the unexpected link to human obesity,^81^, ^105^, ^106^ the characterization of m^6^A readers is of equal importance. These factors represent the direct link between m^6^A and its functional repertoire (Figure 2). YTH domain family members (YTHDF1‐3 and YTHDC1) have been characterized as the first proteins to directly interact with m^6^A‐modified mRNAs.^4^, ^86^, ^107^ The biological roles of these proteins remain largely elusive, as only YTHDF2 has been reported to target m^6^A‐modified transcripts to mRNA decay sites in mammalian cells.^4^ YTHDF2 directly recognizes m^6^A‐modified mRNAs via its carboxy‐terminus and in turn controls the half‐life of the respective mRNA. Interestingly, during yeast meiosis m^6^A might stimulate the translation, rather than mark the degradation, of the respective mRNAs.^108^

In addition, proteins that indirectly read m^6^A have been characterized.^81^ HNRNPC (heterogeneous nuclear ribonucleoprotein C) affects alternative splicing, and the binding of this protein to RNA is stimulated by altered local RNA structures caused through the methylation of adenosine. By influencing the structure of RNA, m^6^A indirectly attracts binding proteins.^4^, ^100^ Additional connections between m^6^A and alternative splicing have also been proposed.^101^

Overall, the impact of m^6^A on RNA is extremely diverse, as this modification has been implicated as a circadian clock pacemaker that facilitates nuclear processing and mRNA export.^102^ Other groups have demonstrated an interplay between m^6^A and ncRNAs, i.e., m^6^A modifications promote primary‐microRNA (pri‐miRNA) processing, and vice versa miRNAs themselves can regulate m^6^A formation.^100^, ^109^, ^110^

However, m^6^A research is still facing a knowledge gap on how modified mRNAs are translated into proteins. Is the ribosome directly affected by m^6^A modifications? If so, which step of translation is targeted? Zhou and colleagues have shown that m^6^A promotes the initiation of translation via the m^6^A reader protein YTHDF2.^98^ In response to heat stress m^6^A methylations within the 5′ UTR of mRNAs are shielded from FTO‐mediated demethylation by the binding of YTHDF2 and facilitate cap‐independent translational initiation. In addition, a single m^6^A residue within the 5′ UTR enabled the translation of an uncapped mRNA,^98^ potentially through the specific binding of the initiation factor eIF3.^111^

Translation initiation is also regulated through YTHDF1.^99^ YTHDF1 selectively reads m^6^A sites located near the 3′ end of mRNAs and promotes the translation of the respective mRNA via an interaction with the ribosomal initiation complex.^99^ Whereas the m^6^A reader proteins YTHDF1 and YTHDF2 both promote translation by facilitating the rate‐limiting step of translational initiation, YTHDF2 also determines the lifetime of an mRNA by chaperoning it to mRNA decay sites.^4^, ^98^, ^99^, ^112^

The role of m^6^A and its interaction with diverse proteins has been extensively studied, but equally interesting are the interactions of modified bases with other nucleotides. m^6^A exclusively base pairs with uridine, indicating that the *N* ^6^‐methyl group does not alter canonical base pairing.^113^ Reverse transcriptase reverts both adenosine and m^6^A to thymine. However, how does the ribosome process an m^6^A‐modified codon?

Initial studies employing methylated mRNAs have reported the stimulation of translation in a rabbit reticulocyte *in vitro* translation system.^114^ However, an increased m^6^A content in mRNAs beyond 5% strongly inhibits translation.^3^ In these reports, neither the amount, nor the positions of the methylation sites were defined. Recent publications applied a systematic approach to analyze the impact of m^6^A on translational elongation in bacterial systems.^115^, ^116^, ^117^ m^6^A was site‐specifically incorporated into the first, second, or third codon position of mRNAs employed for *in vitro* translation systems. Analyzing the protein products revealed codon position‐dependent effects of m^6^A.^116^ Methylated lysine codons (codon triplet: AAA) reduced translation rates, predominantly those with the *N* ^6^‐methyl group present in the first codon position (m^6^AAA). The second (Am^6^AA) and the third codon position (AAm^6^A) were less sensitive to this modification.^115^, ^116^ A recent approach investigating the effects of m^6^A on single steps of translational elongation led to the same conclusions and showed that m^6^A delays tRNA accommodation.^115^ These reports suggest that m^6^A sites might slow ribosomal decoding. Consequently, methylations could reduce protein yield or they might bring protein synthesis into accordance with protein folding or recognition by chaperones.^115^, ^116^

### Pseudouridine

In the early 1950s, prior to the characterization of m^6^A, pseudouridine (Ψ) was isolated from calf liver and initially described as the ‘fifth nucleotide’.^118^, ^119^, ^120^, ^121^ Pseudouridine, i.e., the C^5^‐glycoside isomer of the nucleoside uridine, is formed after the breakage of the *N* ^1^‐glycosidic bond and a 180° rotation of the base through the attachment of the C^5^ atom to the sugar ring. The isomerization does not affect base pairing at the Watson–Crick edge, however, a second hydrogen bond donor is liberated at the Hoogsteen edge that equips Ψ with distinct chemical properties.^122^

**Figure 3:**
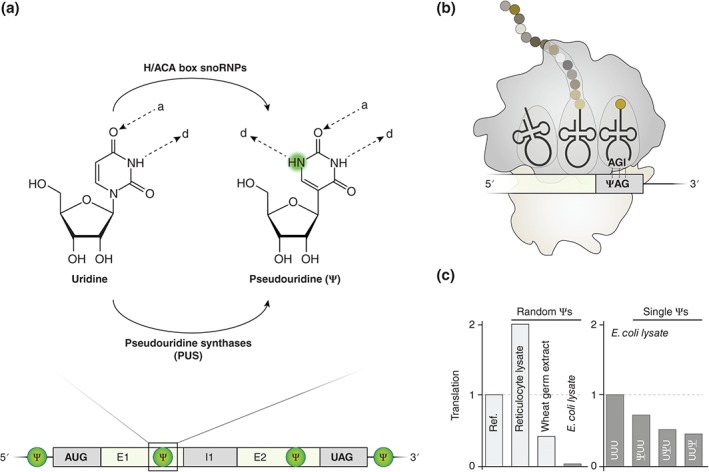
Pseudouridylation directly affects ribosomal translation. (a) Uridine isomerization to Ψ in mRNAs is achieved by two independent mechanisms. Either H/ACA box snoRNAs guide the catalytically active pseudouridine synthase Cbf5/dyskerin to a cognate target sequence, or pseudouridine synthases directly modify a target RNA independent of guide RNAs. Thereby, a second hydrogen bond donor (d) is liberated at the non‐Watson‐Crick edge of Ψ, whereas the Watson–Crick edge is unchanged (a: hydrogen bond acceptor). (b) The pseudouridylation of stop codons leads to stop codon read‐through.^123^, ^124^ In more detail, ΨAG/ΨAA stop codons can be recognized by tRNA^Ser^ or tRNA^Thr^, whereas ΨGA stop codons interact with tRNA^Tyr^ or with tRNA^Phe^ thereby competing with release factors. (c) Ψ interpretation by the elongating ribosome is not universally conserved. Whereas randomly pseudouridylated mRNAs yield higher protein levels in rabbit reticulocyte lysates, translational rates are reduced in wheat germ extracts and are nearly abolished in *E. coli* lysates.^3^ The extent of translational inhibition by single Ψs in bacteria depends on the position of Ψ within a codon (ref: unmodified mRNA).^116^

Generally, Ψ formation is catalyzed by two independent enzymatic reactions (Figure 3(a)). One mechanism to introduce Ψs depends on a subclass of small nucleolar RNAs (snoRNAs), i.e., H/ACA box snoRNAs.^122^ SnoRNAs can be divided into C/D box snoRNAs and H/ACA box snoRNAs, which catalyze the 2′‐O‐methylation and pseudouridylation of cellular RNAs, respectively. These molecules represent a diverse class of nucleolar, intermediated‐sized ncRNAs, found in eukaryotes and archaea.^125^, ^126^ Functional snoRNAs form ribonucleoprotein complexes (RNPs, snoRNPs) and guide catalytically active proteins to the target site via basepairing to the cognate RNA target sequence. In case of H/ACA box snoRNPs, the catalytically active RNP component is the pseudouridine synthase Cbf5/dyskerin.^127^, ^128^, ^129^ The canonical target of a majority of snoRNAs is ribosomal RNA (rRNA), but small nuclear RNAs (snRNAs) are also modified through a distinct population of snoRNAs designated as Cajal body‐specific RNAs (scaRNAs).^130^, ^131^ Interestingly, mRNAs have also been identified as putative snoRNA targets.^132^

In contrast to snoRNPs, the ubiquitous group of pseudouridine synthase (PUS) proteins can modify tRNAs, rRNAs and snRNAs independently of guide RNAs.^133^, ^134^, ^135^, ^136^ Instead, PUS proteins themselves recognize structural and sequence motifs of their target RNAs and perform the pseudouridylation.^133^, ^136^

Ψ is particularly enriched in rRNAs and tRNAs, but is also detected in snRNAs.^7^, ^13^, ^122^, ^137^, ^138^ However, more than 60 years after its initial characterization, several independent groups have also identified Ψ within the mRNAs of eukaryotes.^17^, ^19^, ^20^, ^122^, ^139^ The pseudouridylation of mRNAs has not been previously described because of a lack of effective high‐resolution detection methods. In 2014, three groups conducted Ψ‐selective deep sequencing approaches based on the chemical treatment of RNA with CMC (*N*‐cyclohexyl‐*N* ′‐(2‐morpholinoethyl)carbodiimide metho‐p‐toluenesulfonate) and subsequent reverse transcription. CMC specifically labels Ψs thereby blocking the reverse transcriptase one nucleotide downstream of the Ψ site. These sequencing techniques were designated as Pseudo‐seq, Ψ‐seq and PSI‐seq.^17^, ^19^, ^20^, ^140^ Several hundred Ψ sites in human and/or yeast mRNAs have been revealed with a subset of sites differentially modified in response to stress stimuli. Genetic experiments revealed several Pus proteins and/or snoRNAs as responsible for Ψ formation within mRNAs.^17^, ^19^, ^20^ Subsequently, a refined Ψ profiling method was developed that employed the pre‐enrichment of Ψ‐modified RNAs.^139^ The authors reported thousands of Ψ sites within mammalian mRNAs with a Ψ/U ratio of 0.2–0.6%, consistent with the number of m^6^As within mRNAs.^24^, ^83^, ^84^, ^139^

Ψ formation is dynamically induced in response to environmental cues. However, unlike m^6^A formation, the introduction might not be reversible, as Ψ forms an inert C‐C bond.^141^ Nevertheless, it has been suggested that Ψ plays a global regulatory role. Schwartz and colleagues hypothesized that Ψs stabilize mRNAs or alternatively target the respective transcripts to stress granules during heat stress.^20^, ^142^ Alternatively, Carlile and colleagues suggested Ψ‐induced structural changes to indirectly alter mRNA metabolism.^17^

Karijolich and colleagues investigated the impact of Ψs on translation termination (Figure 3(b)).^123^ A pre‐mature termination codon (PTC) in a reporter mRNA was site‐specifically pseudouridylated employing artificial H/ACA box snoRNAs. The modified stop codon reduced recognition by release factors. Instead of releasing the peptide, a specific aminoacylated tRNA binds to the ribosomal A‐site resulting in a read‐through of the PTC.^123^ ΨAA and ΨAG stop codons resulted in serine and threonine incorporation, whereas ΨGA stop codons encoded tyrosine or phenylalanine.^123^, ^124^ Ψs were not identified to be present in stop codons *in vivo*, and these findings therefore might not be relevant for regulating endogenous translation.^20^ However, Ψ‐dependent stop codon read‐through could be applicable for the development of novel therapeutic approaches targeting pathological PTCs.^143^

It is a longstanding enigma whether Ψ might also interfere with codon recognition during translation elongation. Thus, Ψs could potentially expand the genetic code through recoding translation, i.e., changes in the amino acid composition of the translated peptide, without adjustments in the primary nucleotide sequence of the mRNA.^7^, ^20^, ^138^, ^139^, ^141^, ^144^ This debate was initially stimulated by a report demonstrating that pseudouridylated tRNA anticodons change codon preferences.^145^ Molecular dynamics simulations of Ψ in mRNAs supported the hypothesis of a possible recoding potential through Ψ.^144^ At least in a bacterial *in vitro* translation system, the incorporation of a single Ψ at all three possible positions of the phenylalanine codon (UUU) did not stimulate translational mis‐/recoding based on mass spectrometry of the synthesized peptides.^116^

Whereas the decoding process is not affected by Ψs, the translational rates and protein expression levels increased.^3^ Moreover, HPLC‐purified pseudouridylated mRNAs do not trigger an immune response and are more stable compared with mRNAs containing only uridine.^3^, ^146^, ^147^, ^148^ Karikó and colleagues exploited these Ψ characteristics and injected Ψ‐modified erythropoietin mRNAs into mice. Subsequently, these authors observed 10–100‐fold increased erythropoietin levels compared with translation from U‐containing mRNAs.^149^

Although translation is a highly conserved process, the stimulating effect of Ψ on translation is not universal (Figure 3(c)). The random incorporation of several Ψs in transcripts enhanced translation in mice and in one mammalian *in vitro* translation system, i.e., rabbit reticulocyte lysate.^3^, ^149^ In contrast, in wheat germ translation systems an inhibitory effect was observed.^3^ Several Ψs within an mRNA completely abolished translation in an *E. coli* based *in vitro* translation system, whereas single Ψs did not dramatically change *E. coli* translational rates and kinetics.^3^, ^116^

The mechanisms by which ribosomes interpret Ψs are diverse and are not conserved between the domains of life. Nevertheless, pseudouridylations might be attractive for clinical approaches, reflecting the particular characteristics of these modifications, e.g., for the efficient reprogramming of somatic cells to pluripotency employing Ψ‐modified mRNAs.^150^

After deciphering the Ψ‐transcriptome and numerous sophisticated biochemical studies, Ψ remains an enigmatic mRNA modification, even 65 years after its initial detection.

### 5‐Methylcytosine

5‐Methylcytosine (m^5^C) is not only a well‐characterized DNA modification e.g., reported to be an epigenetic marker in gene regulation and crucial for X‐inactivation, but m^5^C also decorates RNA.^151^, ^152^ Compared with m^6^A and Ψ, little is known about the functions of m^5^C within RNAs. Thus far, m^5^C has been identified in bacterial, archaeal and eukaryal rRNAs, whereas in the latter two domains, tRNAs are also m^5^C‐modified.^153^, ^154^, ^155^ m^5^C has also been reported in ncRNAs and described to regulate their processing.^156^, ^157^

Viral and archaeal mRNAs are subjected to m^5^C modifications.^18^, ^82^, ^158^ Whether eukaryal mRNAs harbor m^5^C or not was a longstanding controversial question in the field. The results of previous studies conducted in the 1970s have been inconclusive, as m^5^C mRNA was detected in HeLa cells^159^ and at low levels in the hamster BHK‐21 cells,^24^ but not in other rodent cell lines, i.e., Novikoff hepatoma^72^ and mouse myeloma cells.^76^

With the rise of bisulfite deep sequencing and its adaptation for RNA research, m^5^C has gained much attention.^157^, ^160^, ^161^ In 2012, a global transcriptome analysis unveiled more than 10,000 m^5^C modification sites within human mRNAs.^161^ The mapped m^5^C pattern is not random, but rather is enriched in the UTRs of mRNAs and in the vicinity of Argonaute binding sites. Squires and colleagues implicated m^5^C in translational regulation, in analogy to m^6^A.^98^, ^99^, ^108^, ^161^ Similar to m^6^A, the deposition of m^5^C appears to be dynamic. However, unlike m^6^A, the methylation has not yet been reported to be fully removed, but is oxidized to 5‐hydroxymethylcytidine (hm^5^C).^162^, ^163^ A recent study demonstrated that mRNAs harboring m^5^Cs are translated *in vitro* at reduced levels, whereas hm^5^C did not affect protein yields. *In vivo,* however, hm^5^C containing mRNAs were associated with polysomes, indicating higher levels of translation.^164^ These results suggest a dynamic, regulatory role of cytosine base modifications. In contrast, earlier studies did not observe an inhibition, but a stimulating effect of m^5^C on translation *in vitro* and *in vivo*.^3^, ^150^ Therefore the influence of m^5^C within coding sequences of mRNAs on eukaryal translation is not yet fully clarified.

The m^5^C modification has not yet been identified within bacterial mRNA. However, employing a bacterial *in vitro* translation system, it was recently demonstrated that single m^5^C modifications do not strongly inhibit protein synthesis independent of their localization within a codon.^116^ Instead, m^5^C induces mis‐/recoding when positioned in the second codon position of a proline codon (Cm^5^CC).^116^ Although the absolute number of mutated peptides was relatively low, the miscoding of Cm^5^CC codons was induced 50‐ to 500‐fold, assuming an endogenous translational error rate of 10^−3^ to 10^−4^.^116^, ^165^, ^166^ Whether this mechanism is biologically relevant to increase protein diversity, such as deamination through RNA editing, needs to be addressed in future studies.

### The Epitranscriptome Is Expanding

In the last decade of RNA research, significant technical advances have been made. With the refinement of next‐generation sequencing^17^, ^19^, ^20^, ^86^, ^161^ and the rise of RNA mass spectrometry,^167^ RNA modifications have re‐gained much attention. Thus, the RNA modification repertoire is constantly expanding and the significance of the RNA modifications involved in several cellular aspects is currently undisputed.

Methylations of the ribose 2′‐OH of mRNA nucleotides within the coding sequence have not unambiguously been identified thus far. However, there are indications that mRNAs are potentially methylated in a snoRNA‐dependent manner. The class of C/D box snoRNAs typically guides a protein complex to the rRNA target, consequently leading to a 2′‐O‐methylation.^168^ However, so‐called orphan snoRNAs have been identified and predicted to target other RNA species, such as mRNAs.^169^ snoRNA SNORD‐115 has been suggested to methylate the pre‐mRNA of 5‐HT_2C_, thereby potentially regulating gene expression.^170^ *In vitro* studies have shown that 2′‐O‐methylations, particularly at the second nucleotide of the codon strongly repress protein synthesis, independent of the sequence context.^116^ This finding suggests that 2′‐O‐methylation is a potent regulator of gene expression at the translation level.

Recently, two independent groups reported *N* ^1^‐methyladenosine (m^1^A) within thousands of the mRNAs of several human and murine cell lines and in yeast.^21^, ^22^ Interestingly, the m^1^A pattern is conserved in these cell types.^22^ Moreover, m^1^A is dynamically deposited in response to environmental cues within 5′ UTRs around canonical and alternative translation initiation sites and in highly structured RNA regions in the vicinity of start codons.^21^, ^22^ m^1^A also affects the structure of RNAs.^22^, ^171^, ^172^ Together with the finding that m^1^A‐modified mRNAs are translated at higher rates compared with non‐methylated mRNAs, the authors hypothesized that m^1^A might affect mRNA folding around the translational initiation sites thereby facilitating translation.^22^ Alternatively, these authors reasoned that m^1^A generates a binding site for proteins, thereby promoting initiation. Overall, the stress‐induced deposition of m^1^A, respectively its reversibility and the proposed implication in translation are reminiscent of m^6^A.

A subset of mRNA modifications (m^1^A, but also m^6^A and Ψ) has been shown to be dynamically regulated and introduced within transcripts in response to stress.^17^, ^20^, ^21^, ^22^, ^98^ Nevertheless, RNA can also be damaged or ‘diversified’ upon excessive stress conditions.^173^, ^174^ The insults, such as radiation, oxidation or damage through chemical agents, can be manifold, harming the RNA integrity.^175^ 8‐oxoguanosine (8‐oxoG), which emerges in oxidized RNAs, and *O* ^6^‐methylguanosine (m^6^G), known as DNA lesion have been recently investigated for their impact on protein synthesis.^174^, ^176^, ^177^ 8‐oxoG hinders tRNA selection and reduces peptide‐bond formation rates, thereby inducing ribosome stalling.^177^ Similarly, m^6^G also affects translation only when present in the second codon position.^174^ These reports indicate that modified nucleotides, as a result of mRNA damage, can severely affect a cell, and that the ribosome is a major target not only of regulatory but also of aberrant mRNA modifications.

## CONCLUSION

The emerging roles of mRNA modifications are extremely diverse, ranging from inducing mRNA decay,^4^ RNA structural alterations or varying protein binding affinities.^95^ RNA modifications have been unveiled in unexpected places in mRNAs, thereby additionally expanding the potential functional repertoire (summarized in Table 1). It will be an exciting and challenging future task to distinguish between meaningful epitranscriptomal marks and silent bystander modifications that simply decorate nucleic acids. Thus, it is crucial to validate data originating from large‐scale sequencing studies through technically independent assays to eradicate sequencing artifacts. A promising technique to depict the modification status of a specific transcript's site has previously been successfully applied to m^6^A‐ and Ψ‐modified RNAs, respectively, but might also be applicable to other RNA modifications.^130^, ^178^

**Table 1:**
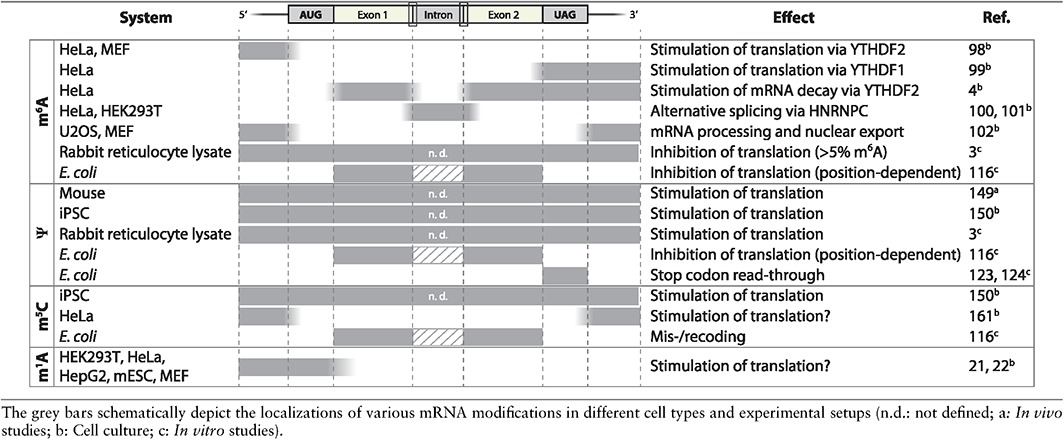
Schematic Overview of Various mRNA Modifications and Their Effect on Gene Expression

It will also be challenging to refine the reported modification patterns to single nucleotide resolution. Sequencing approaches based on immuno‐precipitation narrow down the modification site, but do not precisely map the modified nucleotides. However, improvements of these high‐throughput approaches enable the identifications of some mRNA modifications at single nucleotide resolution.^85^, ^157^, ^161^ Consequently, this will allow refining the modification patterns and will enable the identification of reliable consensus sequences for the entire set of modifying enzymes.

mRNA modifications also modulate protein synthesis (Table 1). Initial studies have indicated that this effect is dependent on the codon position of the modification and in the mRNA sequence context. It will be crucial to define which modified codons directly affect the ribosome as potential regulators of translation. In addition, the mechanism behind this regulatory function will certainly reveal some exciting new insights in the decoding process of modified mRNA nucleotides. Because of the high degree of conservation, it would be expected that all translation systems manage mRNA modifications in similar manner. Nevertheless, contrasting results were obtained, raising a key question: Why is the interpretation of modified codons by the ribosome not universally conserved across different species? It might even be conceivable that within one species, the translational response might vary in different tissues.

RNA modifications were initially described decades ago, whereas the knowledge concerning the presence of these modifications within the coding sequence of mRNAs is rather novel. Thus, investigating the influence of these modifications on pivotal cellular processes, such as mRNA translation, will generate new research opportunities and will change our understanding of gene regulation.
